# Physiological Linkage and Caregiver Mental Health and Well-Being in Dementia Care Dyads

**DOI:** 10.1093/geroni/igaf122.2105

**Published:** 2025-12-31

**Authors:** Kuan-Hua Chen, Casey Brown, Alice Verstaen, James Casey, Jennifer Merrilees, Robert Levenson

**Affiliations:** University of Nebraska Medical Center, Omaha, Nebraska, United States; Georgetown University, Washington, District of Columbia, United States; Lyra Health, Burlingame, California, United States; University of California, Berkeley, Berkeley, California, United States; University of California, San Francisco, San Francisco, California, United States; University of California, Berkeley, Berkeley, California, United States

## Abstract

When people share emotions (e.g., laughing together), their physiological responses can become momentarily “linked” (i.e., changing in coordinated ways)—a phenomenon referred to as “physiological linkage”. Reduced physiological linkage has been observed in social interactions between people with dementia (PWD) and their family caregivers. To expand this work, in two independent samples of caregiver-PWD dyads, we examined the relationship between physiological linkage—measured in laboratory and real-world settings—and caregiver mental health and well-being. In study 1, 64 dyads had a 10-minute conversation about an area of disagreement in the laboratory with six measures of physiological responses continuously monitored. In study 2, 22 dyads wore wristwatch devices in their homes that provided a continuous measure of one physiological measure (activity) over seven days. Physiological linkage was computed as the covariation of dyads’ physiological responses during the conversation (Study 1) or waking hours (Study 2). Caregivers reported their emotional well-being (Study 1) and anxiety symptoms (Study 2) using well-established questionnaires. Findings revealed that lower physiological linkage in the laboratory was associated with lower caregiver emotional well-being (r = 0.27, p = .03). Similarly, lower physiological linkage in the home was associated with higher caregiver anxiety (r = -.48, p = .02). Findings remained statistically significant after accounting for individual-level physiological responses. We believe that caregiver-PWD dyads with lower physiological linkage may have weaker emotional connections, which can increase vulnerability in caregivers to declines in mental health and well-being.

